# The effect of contact needle therapy on fatigue in patients with cancer in palliative care: A study protocol for a randomized controlled trial

**DOI:** 10.1097/MD.0000000000017809

**Published:** 2019-11-01

**Authors:** Keiko Ogawa-Ochiai, Kenichi Yoshimura, Takae Takebe, Mako Iwahashi, Akiko Shirai, Masaki Tsuda, Masao Ogawa, Hideki Ishikawa

**Affiliations:** a Department of Japanese Traditional (Kampo) Medicine, Kanazawa University Hospital, Ishikawa,; b Center for Integrated Medical Research, Hiroshima University Hospital, Hiroshima University, Hiroshima,; c Innovative Clinical Research Center, Kanazawa University Hospital, Ishikawa,; d Mukeido Clinic of Acupuncture, Toyama,; e Department of Anesthesiology, Kanazawa University Hospital, Ishikawa,; f Department of Molecular-Targeting Cancer Prevention, Graduate School of Medical Science, Kyoto Prefectural University of Medicine, Osaka, Japan.

**Keywords:** best supportive care, contact needle therapy, general fatigue, Japanese traditional (Kampo) medicine, randomized controlled trial

## Abstract

**Introduction:**

Almost all patients with end-of-life cancer experience cancer-related fatigue; however, there are only a few known effective coping methods.

**Objectives:**

: We will conduct a prospective, multi-center, single-blinded randomized controlled study to evaluate the efficacy of acupuncture for cancer-related fatigue in patients with end-of-life cancer.

**Methods:**

We will enroll 120 patients with cancer hospitalized in a palliative care unit or receiving consultation from a palliative care team in four hospitals. We will add acupuncture treatment; specifically, contact needle therapy (CNT), consisting of an intervention per week period to the usual care. The primary outcome measure will be the Cancer Fatigue Scale (CFS) score while the secondary outcome measures will be the Numerical Rating Scale (NRS) score for fatigue, pain, and salivary amylase levels.

**Conclusion:**

We will evaluate the possibility of using acupuncture therapy, that is, CNT, in relieving fatigue sensation in patients with advanced cancer.

**Trial registration:** UMIN000028304, registered on July 21st, 2017; https://upload.umin.ac.jp/cgi-open-bin/ctr_e/ctr_view.cgi?recptno=R000032401

## 1 Introduction

Cancer-related fatigue is a distressing, persistent, and subjective sense of physical, emotional, and/or cognitive tiredness or exhaustion related to cancer or cancer treatment and is non-proportional to recent activity and interferes with usual functioning.^[1]^ It has been reported that 100% of patients admitted to the palliative care unit have fatigue complains.^[2]^ Standard therapies for reducing fatigue include exercise, gymnastics, and mental support. Administration of psychostimulants, steroids, carnitine, donepezil, etc, has been suggested as effective pharmacotherapy.^[1,3,4]^ However, exercise therapy and pharmacotherapy are difficult to administer at the end-of-life stage; therefore, fatigue treatment at this stage is difficult.

According to the World Health Organization, acupuncture is used in 129 countries.^[5]^ Many hospitals in the UK have clinically used acupuncture for palliative care of patients at the end-of-life stage.^[6]^ A previous meta-analysis reported that acupuncture is effective for fatigue associated with cancer treatment.^[7]^ However, other than our previously conducted studies, there have been no other studies on the effects of acupuncture on fatigue in end-of-life stage cancer.^[8]^

**Figure 1:**
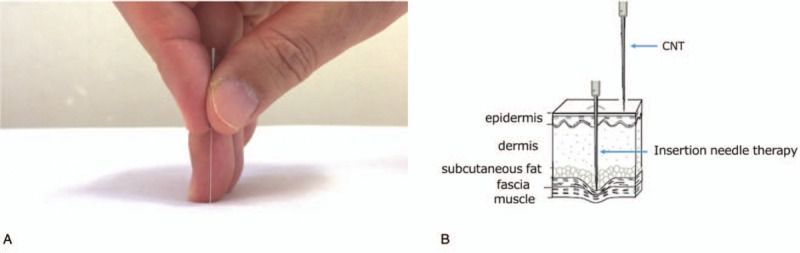
Contact needle therapy (CNT). (A) The method of holding the silver needle for CNT. (B) Diagram depicting the difference between inserted needling and CNT.

The contact needle therapy (CNT) is a needle stimulation technique that is one of the Japanese traditional methods of acupuncture and was developed by Bunkei Ono. It involves the use of disposable silver needles that are not inserted but are only settled on the acupuncture point to provide a minimal but effective stimulus to unblock the meridian (Fig. 1).

A case series assessed the effect of CNT on chemotherapy-induced peripheral neuropathy^[9]^; however, the effect of CNT on general fatigue in end-of-life stage cancer has not been previously evaluated. Therefore, we will evaluate the effect of CNT on general fatigue in patients with end-of-life stage cancer.

## 2 Methods/design

### 2.1 Study design and settings

**Figure 2:**
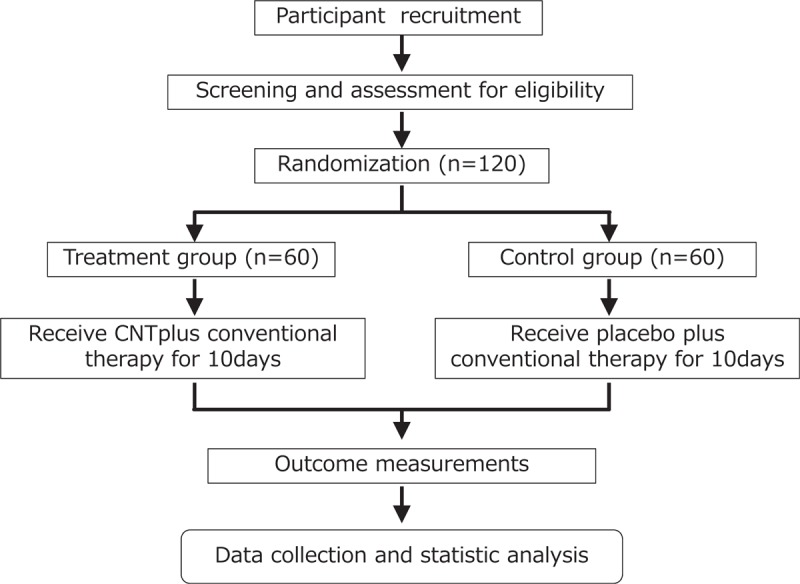
Study design flow chart.

A brief flowchart of the entire study is shown in Figure 2. We will perform a 2-group, randomized, single-blind, placebo-controlled, multi-center trial that will evaluate the efficacy and safety of CNT for palliative care patients with general fatigue. Patients will be recruited from the palliative care departments of 4 hospitals. Informed consent will be obtained from all study participants. The study protocol was designed in accordance with the ethical principles in the Declaration of Helsinki and regional regulations. Central ethical approval for this study has been confirmed from the Central Review Board of Kanazawa University Hospital (ref approval no. 6061) and we will not begin recruiting at other centers in the trial until local ethical approval has been obtained.

This study is registered at https://upload.umin.ac.jp/cgi-open-bin/ctr_e/ctr_view.cgi?recptno=R000032401 (UMIN000028304). The protocol includes elements recommended in the Standard Protocol Items: Recommendations for Interventional Trials^[10]^ checklist (Additional file 1).

### 2.2 Setting and participants

This study will be conducted in Japan. Patients will be recruited from the palliative care inpatient department at each of the hospitals in Hokuriku district.

We will enroll participants based on the following inclusion criteria:

1. 20-years-old and above
2. Cancer patients in the end-of-life stage complaining of fatigue
3. Having terminated treatment
4. Provision of written informed consent for participation after receiving sufficient explanation for participation in this study and achieving sufficient understanding.

Participants who meet the following exclusion criteria will not be enrolled:

1. Patients with metal allergies
2. Inappropriate for study inclusion as per a doctor's judgment.

### 2.3 Randomization, allocation concealment, and blinding

The web-based online randomization system to be used in this trial was provided by an independent academic data management center at Kyoto Prefectural University of Medicine. Enrolled patients who provide informed consent will be blindly randomized (1:1 allocation ratio) into either an experimental treatment group receiving CNT or a control group receiving placebo CNT. All participants and investigators other than acupuncturists will be blinded to treatment allocation.

### 2.4 Interventions

We will perform CNT or sham CNT once a week for 4 weeks. Sham CNT will be performed as an only-hands procedure (the hand that supports the acupuncture). Treatment will begin immediately after obtaining informed consent and web-based randomization.

CNT will be performed on randomly selected cases by acupuncturists with enough training experience and according to the medical diagnosis of meridian therapy. We will use the following acupuncture points:

1. Points for all patients: CV12, CV4, ST25, and KI2.
2. Selected points: LR8, LR14, SP3, LR13, LU9, LU1, KI7, GB25, PC7, CV17, CV6, CV4, ST36, and LU1.

Treatment will be performed once a week for a total of 4 weeks at approximately the same time in the afternoon. No restrictions will be imposed on the standard treatments for pain or any other disease during this study. If the patient is discharged according to the clinical evolution or is deceased, he/she will be excluded from this study. Further, occurrence of a serious adverse event or withdrawal of consent will result to study exclusion.

### 2.5 Data collection

The study data collection process is outlined in Table 1.

**Table 1:**
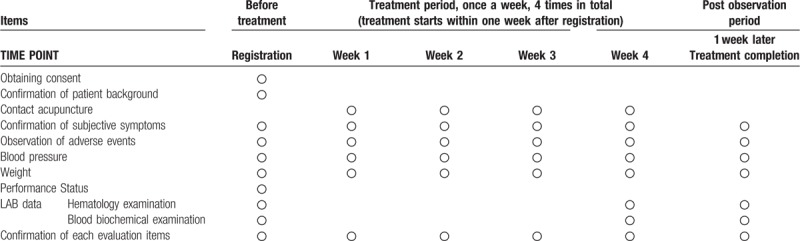
Treatment schedule and outcome measures.

### 2.6 Primary endpoint

Fatigue will be measured using the Numerical Rating Scale (NRS). The primary endpoint will be the NRS-measured fatigue scale value after 4 weeks of treatment. For patients whose treatment will be discontinued, the value measured after the last treatment will be used.

### 2.7 Secondary endpoints

1. Fatigue scale value measured by the Cancer Fatigue Scale (CFS)^[11]^
  The CFS has good stability (average test-retest reliability *r* = 0.69, *P* < .001) and good internal consistency (Cronbach's alpha coefficient for all 15 items = 0.88), which indicates it as a brief, valid, and feasible measure of fatigue for use with cancer patients.
2. Pain scale value measured using the Numerical Rating Scale (NRS)
3. Support Team Assessment Schedule Symptom version Japanese version (symptom) value
4. Palliative Prognostic Index
5. Palliative Performance Scale value
6. Measure salivary amylase activity
  Estimation of stress degree by measuring amylase activity in saliva using a device (Lase Monitor, Nipro) will be performed before and after acupuncture.
7. Adverse events (types and frequency of side effects): Side effects will be recorded for each observed adverse event and the type and frequency will be determined.

### 2.8 Statistical consideration

#### 2.8.1 Sample size

Based on the results of our previous study,^[8]^ we assumed 6.88 and 5.46 as the mean of the NRS-measured fatigue scale value for each group and 2.7 as its standard deviation. Using a 2-sided significance level of 5% and a power of 80%, the required number of patients in each group is 57. Considering dropout cases, we will enroll a total of 120 patients in this study.

#### 2.8.2 Analysis set

The Full Analysis Set will be used for all primary analysis. Per Protocol Set analysis will be performed for evaluating the sensitivity of results.

#### 2.8.3 Statistical analysis

The analysis of covariance will be used to perform between-group comparisons of the NRS-measured fatigue score with the baseline fatigue score of each patient at enrollment as the covariate and a 2-sided significance level of 0.05. Between-group comparisons of secondary endpoints will be performed in the same manner. Regarding secondary endpoints, the evaluation will be performed in an exploratory manner with no adjustments for multiplicity.

### 2.9 Quality control and trial management

The management structure will comprise the principal investigator (PI), a trial management group, and a data monitoring committee. The trial management group will be responsible for conducting the trial and will meet monthly to discuss the trial progress. The PI will visit each collaborative hospital for face-to-face meetings and to share information to promote patient recruitment. The data monitoring committee will review safety and efficacy data. All data will be monitored every month through a central monitoring method. Additional monitoring may be performed at the discretion of the monitoring manager. The data monitoring committee have met once prior to the start of patient recruitment. At least twice per year, participating investigators, research assistants, and research nurses will be required to attend a training workshop on clinical research to ensure strict adherence to the study protocol and familiarity with the trial administration process. The data collected in this trial will comprise information recorded in case report forms and questionnaires. Data quality will be checked regularly by research assistants and overseen by monitors; all modifications will be marked on case report forms and data managers will recheck the data before they are officially logged. The database will be locked after all data have been cleaned. If participants withdraw from the trial during the study period, the reasons will be documented and the dropout rate will be statistically analyzed.

## 3 Discussion

Kampo (Japanese traditional) medicine is the most frequently used alternative and complementary medicine in Japan and includes herbal therapy, acupuncture, and acupressure. CNT is one of the Japanese traditional methods of acupuncture and was developed by Bunkei Ono. Needles are not inserted; rather, they are settled on the acupuncture point to provide a minimal but effective stimulus to unblock the meridian. The aim of CNT is to improve the patient's condition regardless of the underlying disease by regulating the flow of Qi. This method has many advantages; namely, it is safe, painless, easy to perform, and has a low risk of infection. Since ancient times, it has been stated that the larger the needle and the deeper it is inserted, the stronger the stimulation. If the stimulation is too strong, the patient's condition worsens, especially if their constitution is weak. CNT is known as a method of weak stimulation. Given these attributes, CNT is effective and appropriate for treating cancer patients

In our previous study, we reported that although there was no significant change in the CFS, there was a significant decrease in the fatigue NRS values and salivary amylase levels after treatment. This indicates the possibility of using acupuncture therapy to relieve fatigue in patients with advanced cancer.

This study will be single-blinded because the acupuncturists performing the treatment will be aware of the assignment. We will compare the group that receives acupuncture treatment, that is CNT, and the group receives sham CNT, that is, only placing the hand that supports the acupuncture needle.

Fatigue is one of the difficult symptoms to control in palliative care. We may be able to obtain alternative therapy with relatively low risk from this study.

## 4 Trial status

Recruitment began in January 2018 and is expected to be completed in May 2020.

## Acknowledgments

We thank Ms. Mie Morikawa and Ms. Aya Aoyama for their assistance.

## Author contributions

KO, KY, AS, MO, and HI designed the study protocol and drafted the manuscript. MT reviewed the study protocol and drafted the manuscript. KY is responsible for the statistical design and analysis as trial statistician. All authors carefully read and approved the final version of the manuscript.
